# Influence of the Topography of Zirconium Treated with Laser Micropatterning on Periodontal Ligament Stem Cells: An In Vitro Study

**DOI:** 10.3390/jfb17030132

**Published:** 2026-03-09

**Authors:** Ildefonso Serrano-Belmonte, Alba Rico-Molina, Juan Ignacio Rosales-Leal, Guillermo Lorite-Méndez, Miguel Ángel Rodríguez-Valverde, Clara Serna-Muñoz, Ascensión Martínez-Cánovas

**Affiliations:** 1 School of Dentistry, Faculty of Medicine, Clínica Odontológica Universitaria, 2 Planta, Hospital Morales Meseguer, Avda. Marqués de los Vélez s/n, 30008 Murcia, Spain; alba.ricom@um.es (A.R.-M.); clara.serna@um.es (C.S.-M.); ascension.martinez4@um.es (A.M.-C.); 2 Faculty of Dentistry, University of Granada, 18071 Granada, Spain; irosales@ugr.es; 3 Applied Physics Department, School of Science, University of Granada, 18071 Granada, Spain; loriteguille@go.ugr.es (G.L.-M.); marodri@ugr.es (M.Á.R.-V.)

**Keywords:** zirconium, implant abutment, laser micropatterning, periodontal ligament stem cells, initial adhesion

## Abstract

Zirconium is a widely used material in the field of dentistry, employed for implants and their components as well as for the creation of crowns and veneers. Given that its biocompatibility has been studied and demonstrated in various fields of application, it is necessary to analyze how surface modification of this material influences its properties. The purpose of this study was to analyze the biocompatibility, initial adhesion (48 h), and morphology of periodontal ligament stem cells (PDLSCs) seeded on different zirconium surfaces treated with laser micropatterning, as well as plastic coverslips as a control. The Neubauer chamber was used to count the cells adhered to each of the sets, and confocal and scanning electron microscopy were employed to examine the adhesion and morphology of periodontal ligament stem cells on each of the zirconium surfaces studied. Results: Statistically significant differences were found in terms of primary cell adhesion, with sets 3 (grid topography) and 4 (channel topography) showing the most favorable characteristics for fibroblast adhesion. It was concluded that regular and moderately rough surfaces promoted better cell proliferation and development.

## 1. Introduction

In dental restorations carried out by placing implants, the transmucosal component/abutment or transepithelial abutment exerts a significant influence on their mucointegration capacity. Mechanical variations in these abutments, such as their angulation, height, and macrogeometry, as well as biological qualities, have important effects on this interaction with the soft tissues surrounding the implant. Therefore, there has been a tendency to investigate methods that change properties such as topography, roughness, chemistry, loading, and hydrophilicity in titanium-based implants and attachments, but there is little evidence for other materials, of which zirconium, a bioinert ceramic, is the main one [1,2,3,4,5,6]. The biocompatibility of this material has been studied and demonstrated in numerous works [7], but not so much the improvements or disadvantages that may arise from the modification of the surface of zirconium by various methods. Implants and their abutments have evolved from smooth surfaces to ones with a topography more favorable for cell adhesion [8]. It is necessary to study the behavior of the tissues that surround it once it is placed as an implant adjunct. An important part of understanding this behavior is given by the adhesion of the cells that make up this tissue. Laser treatment of these titanium surfaces is one of the mechanical procedures that aim to achieve a nanostructure to which cells can adhere [9,10]. One of the most widely used surface coatings is zirconium, which improves osseointegration, in addition to bioactive ceramics and materials such as hydroxyapatite. Numerous studies have shown that microscale regular surfaces treated with bioactive elements are the most suitable for facilitating this adhesion [11,12,13]. There have been studies that have analyzed the behavior of stem cells derived from dental pulp on the implant surface with respect to adhesion [14]. Much research has been done on osseointegration and cell adhesion affected by the surface properties of the titanium dental implant itself. Other studies have shown the effect of laser surface treatment on clinical attachment levels and crestal bone remodeling around implants with immediate functional loading [15]. However, the influence on implant attachments has not been studied in such depth, especially on zirconium attachments [4]. The development of new surfaces with optimized topography can lead to better interaction between cells and materials used in implantology [16]. When a cell begins the adhesion process, a series of changes occur in its morphology, generating extensions of the cytoskeleton that allow it to move on the substrate, causing the cell to increase its volume and obtain a more elongated shape. These extensions are called lamellipodia and filopodia. Lamellipodia are broad, flattened extensions, while filopodia are thinner protrusions. They are dynamic membrane structures that guide cell movement and substrate interaction, respectively. Lamellipodia are dynamic extensions that originate from the anterior portion of the cell and direct cell movement through interaction with the actin cytoskeleton and promote adhesion to the extracellular matrix (ECM), while filopodia are involved in microenvironment sensing and guiding cell movement [17,18,19,20].

Periapical tissues contain various mesenchymal stem cells (MSCs), such as periodontal ligament stem cells (PDLSCs), which have demonstrated the ability to differentiate into specialized cell lineages, such as osteoblasts, cementoblasts, adipocytes, and chondrocytes, depending on the inductive culture media used. These cells can therefore form alveolar bone, cementum, gingiva, periodontal ligaments, and even peripheral nerves and blood vessels in vivo. The presence of periodontal ligament stem cells in periapical tissues makes them ideal for analyzing their behavior on materials used in implantology.

The main objective of this study was to analyze the biocompatibility of a type of surface treatment, such as laser micropatterning, and its ability to generate proliferation and changes in the morphology of cells that are part of the periodontal ligament. A null hypothesis can be formulated in which laser micropatterning of zirconium surfaces does not significantly affect the adhesion, viability and apoptosis of periodontal ligament stem cells grown on such surfaces.

## 2. Materials and Methods

The material used to carry out the study is monolithic zirconium, Cercon Base (Dentsply Sirona GmbH, Bensheim, Germany). The samples were sintered and washed with deionized distilled water. A micro-pattern of the surface was created with a laser (Rofin RSY E-20 SHG II, Hamburg, Germany) that gave rise to four groups:

Group 1 (SET 1: *n* = 9) peak topography, laser width of 10 μm, periodicity of 15 μm, laser power of 22 A, intensity of 15,000 Hz, and velocity of 100 mm/s.

Group 2 (SET 2: *n* = 9) smooth topography with random irregularities; control group.

Group 3 (SET 3: *n* = 9) grid topography, laser width of 10 μm, periodicity of 60 μm, laser power of 22 A, intensity of 15,000 Hz, and speed of 100 mm/s.

Group 4 (SET 4: *n* = 9) channel topography, laser width of 10 μm, periodicity of 15 μm, laser power of 22 A, intensity of 15,000 Hz, and speed of 100 mm/s.

After micro-patterning, the samples were heated to 1000 °C for 1 h and allowed to cool for 9 h. The samples were then calibrated to give values of 0.75 cm (depth) × 0.75 cm (width) × 0.16 cm (height) for each of them.

Each of the samples went through the following process: immersion in pure ethanol, drying, immersion in distilled water, drying and bagging, and sterilization in an autoclave (Vacuklav 24 B/L+, MELAG, Berlin, Germany) using a Universal Program at 134 °C.

Along with the 36 samples, another 9 samples were used as a positive control (P5: *n* = 9), using sterile plastic coverslips for tissue culture, 13 mm in diameter, with surface treatment for cell adhesion (Sarstedt, Inc., Nümbrecht, Germany).

Human periodontal ligament was obtained from impacted third molars from 14 healthy volunteers who provided written informed consent, as indicated by the Ethics Committee of the University of Murcia (CBE 599), which had been extracted for orthodontic reasons. To obtain the cells, the periodontal ligament was isolated and immersed in 3 mg/mL of type I collagenase (Sigma-Aldrich, Saint Louis, MO, USA) for 1 h at 37 °C. Cells were seeded in two 25 cm^2^ plastic tissue culture flasks (BD Biosciences, San Jose, CA, USA) at 37 °C with 5% CO_2_ for three days. Erythrocytes and other non-adherent cells were then removed, and growth medium was added. Adherent cells proliferated to 80% confluence and were identified as zero-pass cells (P0), after which the mesenchymal phenotype of the cells was confirmed by observing their mesenchymal markers. Prior to their use in the in vitro experimentation, the PDLSCs were characterized to confirm their mesenchymal nature. The cells were analyzed via flow cytometry (FACSCalibur flow cytometry system; BD Biosciences), confirming high expression of the mesenchymal stem cell (MSC)-specific surface markers CD73, CD90, and CD105, as well as low expression of the hematopoietic markers CD34, CD45, CD14, and CD20 [21]. The PDLSCs were frozen in liquid nitrogen at −196 °C.

At the start of the materials study, cells were cultured in Dulbecco modified Eagle medium with 4.5 g/L of glucose and phenol red supplemented with 10% fetal bovine serum (SBF), 4 mM glutamine, and 1% penicillin/streptomycin in two 75 cm^2^ and one 25 cm^2^ culture flasks and incubated at 37 °C, 7.5% CO_2_, and 85% relative humidity. Three subcultures with a medium change four days after each were required to obtain the appropriate number of cells. Periodontal ligament cells were seeded in 45 wells with a density of 12850 cells/cm^2^, and culture conditions were maintained.

Thus, all samples were cultured for 48 h and subdivided into sets of three within each group of material (Table 1).

Cell proliferation was measured using the trypan blue viability assay. After culturing the cells and after the incubation period (in this case, 24 and 48 h), the samples were separated into a different well from the medium in which they were immersed. This was done to determine the number of cells attached to the sample and the number of cells suspended in the medium. The cells were washed with 5 mL of PBS to weaken their adherence to the well, and 300 μL of 0.25% trypsin was added to the well, swirling horizontally to cover the entire surface. The trypsin was aspirated, leaving a small volume. The flask containing the cell line was then incubated with trypsin for 5 min at 37 °C. Five minutes later, under the microscope, the cells appeared round and motile, having lost their adherence. A total of 400 μL of DMEM was diluted in the trypsinized culture flask, along with an additional 400 μL of cleaning medium. The suspension was pipetted twice to further lift the cells, and 100 μL of 0.4% trypan blue was added to a well of a multiplate. The cell suspension (in culture medium) was homogenized, and 100 μL was added to the microtube containing trypan blue. A total of 10 μL of the mixture was placed in each chamber of the hemocytometer, and the sample was observed under a microscope. The blue (dead) cells and the birefringent or white (live) cells observed in each of the squares were counted separately in the Neubauer chamber.

To morphologically assess cell apoptosis, Hoechst 33342 was used to label the nuclei at a final concentration of 1 μg/mL. Each sample was imaged with a Stellaris 8 Confocal Laser Scanning Microscope (Leica Microsystems, Wetzlar, Germany) at a scale of 50 μm. The images were entered into the Image J-win 64 program, the FIJI macro developed by the image service of the University of Murcia was used, and the “Cell Counter” plug-in developed by Kurt de Vos was used. Cell death was assessed by counting at least 200 cells of each concentration. After the count carried out by the program, the apoptotic and necrotic cells were discarded to later calculate the percentage of living and dead cells.

**Figure 1:**
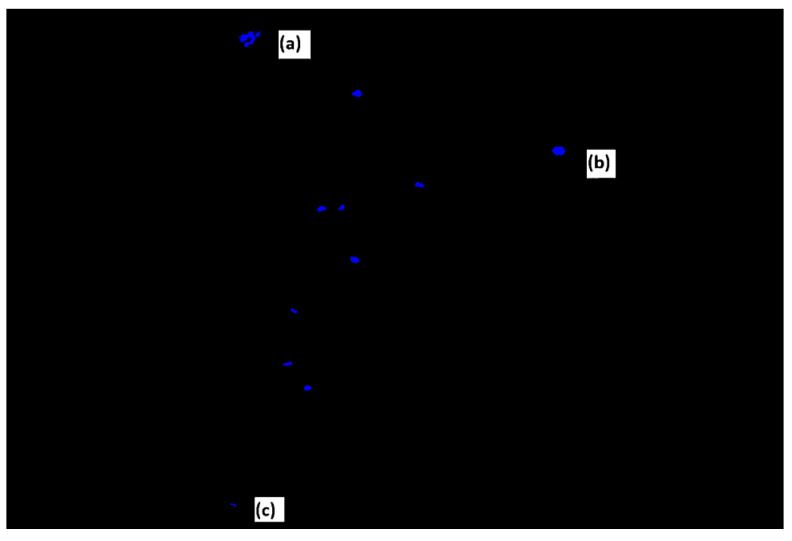
Image of the confocal microscope with the Hoescht 33342 stain. (**a**) Apoptotic cell, (**b**) normal cell, and (**c**) necrotic cell.

Live cells exhibit normal morphology and low, homogeneous blue fluorescence. In contrast, dead cells exhibit a different morphology and intense blue fluorescence (Figure 1). Using this stain, pyknotic and apoptotic nuclei can be differentiated.

After 48 h of incubation, the culture medium was removed, and the cells were fixed with 2.5% glutaraldehyde for 20 min. The samples were immersed in a scrubber composed of 0.2 M cacodilat buffer, distilled water and sucrose to preserve them. After being fixed, they were dehydrated in acetone with increasing concentrations (50%, 70%, and 90% for 10 min and 100% in two batches of 10 min) and then dried at a critical point. Once dry, the samples were assembled and coated with platinum (5 nm). Images were taken with an ApreoS Field Emission Scanning Electron Microscope (FESEM) (Thermo Scientific, Waltham, MA, USA) of each of the 5 groups at a magnification of 300× to analyze the area of cells occupied by the surface. In addition, several images were taken at a magnification of 600× and 1200× to analyze the morphology of the cells in each of the samples. The images were entered into the Image J-win 64 program, the FIJI macro developed by the University of Murcia was used, and the “Cell Counter Threshold-Yen” plugin was used, in which, after pointing out the non-useful areas, the % of occupied area of the different zirconium sets was analyzed. The “Cell Counter Manual” plug-in was used for the control samples of the plastic discs, in which the surface that was with cells had to be manually marked.

The statistical data were analyzed with the SPSS^®^ software package (version 22.0) for Windows. The Kruskal–Wallis test was used to analyze cell viability and apoptosis or death, and to assess the area of cells occupied by the surface, which allows a non-parametric comparison of the data obtained across the different sets. In addition, the Dunn–Bonferroni test was also applied to make two-to-two comparisons between them (α = 0.05 for all tests).

## 3. Results

**Figure 2:**
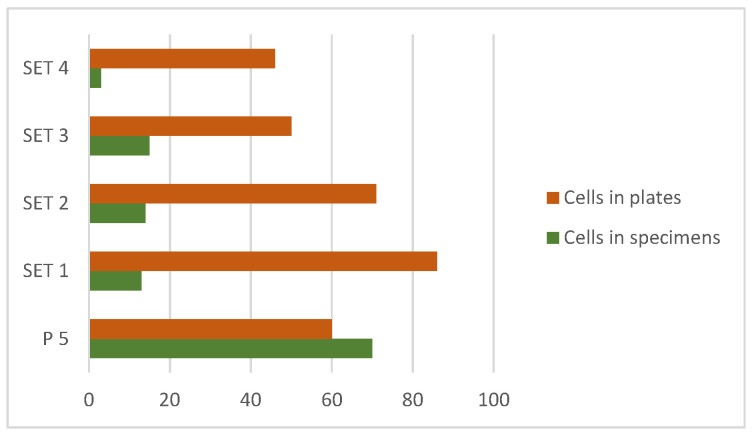
Difference between the number of cells attached to the specimens and the number of cells remaining in the medium of the plates (48 h).

First, a descriptive analysis of cell viability was carried out regarding the material itself, observing that the values of this count are in a range of 1 to 32 cells. Carrying out the Kruskal–Wallis test, a significant difference was found between the control sample (P5) (*p* = 0.0364), but not between the different sets. Analyzing the median of each of them, it was observed that set 3 registered a higher number of cells. On the other hand, looking at the median of set 4, it has been observed that this is the material that deviates the most from the pattern followed by the rest, since it is the one that registered the lowest number of cells; even so, it still does not differ statistically significantly (Figure 2).

On the other hand, regarding the descriptive analysis of cell viability in the wells, the counts were between 8 and 38 cells. Using the latter test again to carry out a comparison, no significant differences were observed either between the sets with each other or with the control. The only set whose mean and median were even higher than the control sample was set 1.

**Figure 3:**
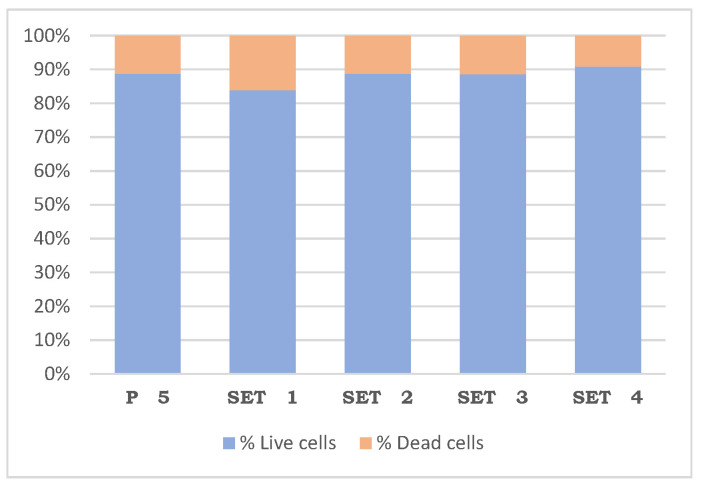
Percentage of live and dead cells (48 h).

In the apoptosis or cell death assay, a non-parametric contrast of the results obtained was carried out by applying the Kruskal–Wallis test, which allowed us to compare the four sets and the control with each other. In the comparison, no statistically significant difference was found, including between the control and between the sets themselves, in terms of the percentage of live and dead cells. It was only observed that, performing a two-to-two comparison between the sets and the control using the Dunn–Bonferroni test, a statistically significant variation appears between sets 4 and 1 (*p* = 0.0271), with set 1 having the lowest % of both living and dead cells (Table 2), (Figure 3).

Regarding the percentage of occupied area test, a non-parametric contrast of the data was also performed, applying the Kruskal–Wallis test to carry out a comparison of the four sets and the control with each other. Statistically significant differences were observed both when the control sample was included (*p* = 0.0001) and when it was not (*p* = 0.0035). A greater difference was observed between sets 1 and 3 due to greater variability between their medians. Therefore, a two-to-two comparison was also carried out between each of the sets by applying the Dunn–Bonferroni test (Table 3).

**Figure 4:**
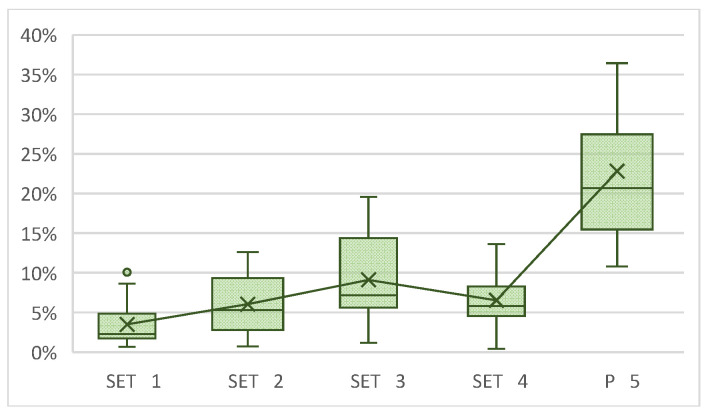
Percentage of occupied area (48 h).

The table shows the two-to-two comparison between each of the sets (not including P5). As mentioned above, the most statistically significant difference is found between sets 1 and 3 (*p* = 0.0002). Statistically significant variations were also observed between sets 2 and 1 (*p* = 0.0279) and between sets 4 and 1 (*p* = 0.0081) (Figure 4).

**Figure 5:**
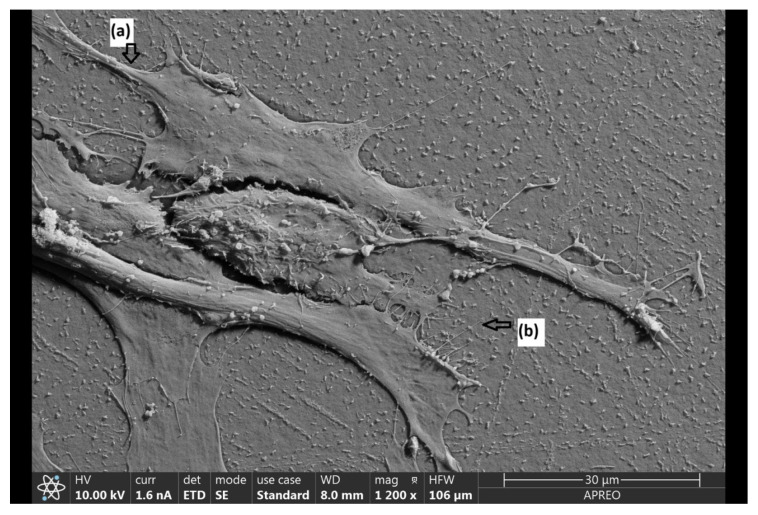
Parts of the fibroblast during adhesion (1200×). (**a**) Lamellipodia; (**b**) filopodia.

Initially, when fibroblasts contact a material, they have a predominantly round shape. However, over time, they acquire a more starry or fusiform morphology, extending along the material, and develop a series of prolongations (filopodia and lamellipodia) that allow them to join with other cells (Figure 5).

**Figure 6:**
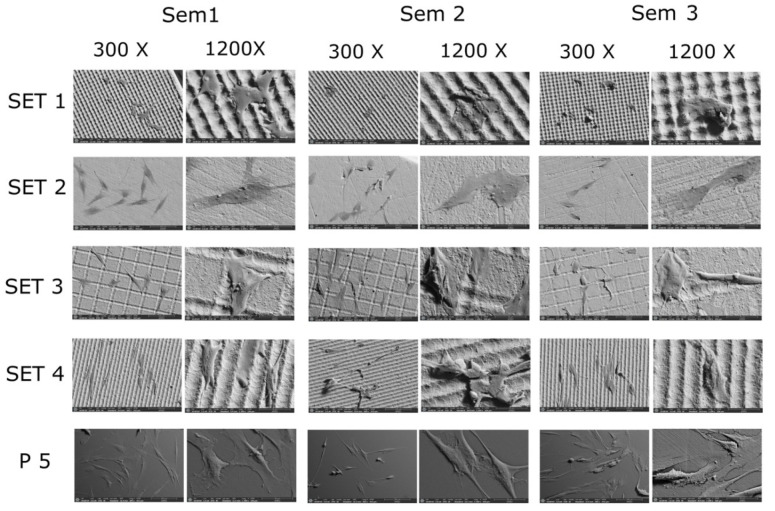
Photographs at lower and higher magnification of each of the sets using FESEM. Sem corresponds to each of the samples observed under the microscope.

The cell morphology of the different samples was observed with the Field Emission Scanning Electron Microscope, where it was found that, even if they remained at the same time and under the same conditions, the development of the cells after 48 h varied significantly (Figure 6).

Set 1: The peak topography of this sample seems to be the least favorable for the growth of fibroblasts since they have acquired a more geometric morphology, which would indicate that the surface does not seem to allow them adequate expansion and adhesion.

Set 2: In this sample, whose topography is smooth with random irregularities, which is considered the control sample of the zirconium itself, an advanced growth of fibroblasts is observed with a greater number of these than in the other sets, being more flattened, although with more random growth compared to the two previous zirconium samples.

Set 3: The grid topography of this sample also allows for better development of the cells, since, as in the previous sample, they seem to follow the perpendicular channels that make up the grid.

Set 4: The canal topography presented in this sample seems to favor the growth and expansion of fibroblasts, as they have acquired a more elongated shape and are developing filopodia and lamellipodia along these channels.

Plastic coverslips: Growth is noticeably greater because they are disks whose surfaces have been specifically treated to promote greater cell adhesion, which is why they were used as a total control.

Looking at the microscope images shown below, we can see that the PDLSCs that had the greatest development were those grown on the polystyrene discs (the sample that we used as a total control), followed by set 4, set 3, set 2 and finally set 1, as described above.

## 4. Discussion

Despite the complexity of interactions between transepithelial and periodontal ligament cells in vivo, in vitro studies can provide significant information about cell/biomaterial interactions and their mechanisms, such as surface impact [20]. In this research, a feasibility analysis was carried out using the trypan blue assay, while in other studies, such as that by Rizo-Gorrita et al. [17], an MTT assay was carried out, where cells were observed in a spectrophotometer. Both in the latter and in the present research, statistically significant results were obtained at 48 h, used for each of the studies, but not among the samples of each other. Numerous studies have analyzed the adhesion of cells, such as osteoblasts and PDLSCs, to different materials (polystyrene, conventional ceramics, zirconium, titanium, etc.) used in the manufacture of implants, allowing us to compare how the morphology of the surface influences their growth [20]. This is a controversial issue, since some authors agree that smooth surfaces promote better cell development [22,23,24], while others conclude the opposite [25]. On the other hand, studies such as those by Campos-Bijit et al. [26], Frías-Martínez et al. [27], and Canullo et al. [5] have shown that moderately rough topography seems to be the most favorable in terms of cell adhesion, while those that are smooth or extremely rough can hinder cell adhesion. The study carried out by Delgado [28], like the present study, compares cell proliferation on both smooth and machined surfaces. It concludes that machined surfaces with 50 and 100 μm grooves are the most suitable candidates to improve the biological seal at the neck level of the implants, since they stimulate the adhesion and activation of human fibroblasts in this area. For this reason, observing the development of PDLSCs in this research, and in accordance with this latest study, it should be noted that those grown in the samples with channel and/or grid topography obtained better results in terms of their adhesion and growth.

With regard to initial adhesion, numerous studies, such as those by Takamori et al. [11], Yajing Liang et al. [24], Luis M. Delgado et al. [28], Yang Yang et al. [29], and Akashi et al. [30], have carried out an analysis of this at 24 h, or even less (3, 4, 6 and 9 h), obtaining statistically significant results on the proliferation and adhesion of fibroblasts to materials, such as zirconium and titanium. On the other hand, some of the latter and other studies, such as those by Vanessa Campos-Bijit et al. [26] and Luigi Canullo et al. [5], in addition to evaluating adhesion at 24 h, prolonged this analysis over time, observing the adhesion of these cells at 2, 3, 5, and 7 days, with remarkably favorable results in terms of the morphological development of the adhered fibroblasts.

In the present research, it was decided to mainly analyze the proliferation and adhesion of PDLSCs at 48 h, although there are studies that affirm a representative initial adhesion at 24 h; this seems to be more appreciable with the passage of time. Thus, we were able to observe relevant data with the SEM after comparing the area of cells occupied by the surface between each of the different sets. As mentioned above in the results, we obtained statistically representative differences, especially between sets 1 and 3 (*p* = 0.0002), which again demonstrates that moderately rough surfaces are better than smooth or extremely rough ones.

With regard to the study of cell adhesion through the analysis of apoptosis or cell death, certain studies, such as those by Campos-Bijit et al. [26], Yang Yang et al. [29], Braber et al. [31], Canullo et al. [5], and María Rizo Gorrita et al. [17], among others, have analyzed (by means of laser scanning confocal microscopy) how cells adhere to implants 48 h after their surfaces are treated in different ways. This study concludes that regular and moderately rough surfaces, compared to those that are irregular, seem to positively influence cell adhesion. In the results of this research, in general terms, the treated surfaces that present a regular pattern and a certain roughness, such as set 4 (channel topography), present statistically significant differences compared to extremely rough surfaces, such as set 1 (peak topography). On the other hand, studies such as that by Liang et al. [23] demonstrated the impact of zirconium surface treatments, specifically sanding and polishing, on the adhesion and proliferation of human gingival fibroblasts (HGFs), revealing that smoother surfaces promote better cell adhesion and proliferation. In particular, polishing treatment establishes better interactions between cells and materials. Other studies, such as that by Yoshihiko Akashi et al. [30], reported no significant differences in the roughness of the zirconium discs. In this study, there were no differences in the contact angle of the zirconium discs before and after polishing. However, the contact angle of the zirconium discs was lower in the excimer laser irradiation group compared to the non-irradiation group, and wettability improved, thus favoring fibroblast adhesion. The present study by itself presents a series of limitations that can bias our results, one of which we have already commented on previously, and that is the scarcity of contact time between the cells and the material, as we have indicated that a longer contact time can directly improve or decrease the adhesion of our cells.

Another factor to consider is that changes in surface morphology have been studied in this research, but there are several other factors that interfere with this variant to modify cell adhesion. Studies suggest that these factors to be considered are topography (within it the fractal dimension, morphology and roughness), surface chemistry and wettability [32], also proving that by increasing the energy of the surface and, consequently, its hydrophilicity, the interaction between the implant and the biological environment can improve cell adhesion [4].

Despite the limitations already mentioned, this in vitro study has highlighted the ability of regular and moderately rough surfaces to promote the adhesion and distribution of fibroblasts. This suggests that the treatment of zirconium transepithelial surfaces by laser micropatterning has potential biological benefits in terms of cell proliferation and adhesion in early stages. Although this research provides information about the initial adhesion between zirconium and periodontal ligament cells, more studies, including more parameters and a larger number of samples, are needed to obtain more significant results.

## 5. Conclusions

Based on the findings of this in vitro study, the following conclusions were drawn:

1. Laser micropatterning of zirconium surfaces significantly affects the adhesion, viability and apoptosis of periodontal ligament stem cells grown on these surfaces.
2. Adhesion and viability are better on zirconium surfaces, both smooth and with random irregularities such as channels and grids, compared to samples with peak patterning.
3. Apoptosis and cell death were reduced for the channel-shaped micropattern.

## Figures and Tables

**Table 1:** Number of material plates intended for each test. CM: Confocal Laser Scanning Microscope, FESEM: Field Emission Scanning Electron Microscope.

SAMPLE GROUPS		Cell Counting	CM	FESEM
	SET 1 (*n* = 9)	3	3	3
	SET 2 (*n* = 9)	3	3	3
	SET 3 (*n* = 9)	3	3	3
	SET 4 (*n* = 9)	3	3	3
	P5 (polystyrene discs) (*n* = 9)	3	3	3

**Table 2:** Dunn–Bonferroni test comparing each set, two by two, with each other and with total control (*P)*.

	SET 1	SET 2	SET 3	SET 4
**SET 2**	0.0837			
**SET 3**	0.0714	0.4664		
**SET 4**	0.0271	0.2930	0.3227	
**P5**	0.0987	0.4633	0.4300	0.2622

**Table 3:** Dunn–Bonferroni test comparing each of the sets, two by two (*P*).

	SET 1	SET 2	SET 3
**SET 2**	0.0279		
**SET 3**	0.0002	0.0584	
**SET 4**	0.0081	0.3116	0.1408

## Data Availability

The original contributions presented in the study are included in the article, further inquiries can be directed to the corresponding author.
